# Combining Phylogenetic Profiling-Based and Machine Learning-Based Techniques to Predict Functional Related Proteins

**DOI:** 10.1371/journal.pone.0075940

**Published:** 2013-09-19

**Authors:** Tzu-Wen Lin, Jian-Wei Wu, Darby Tien-Hao Chang

**Affiliations:** Department of Electrical Engineering, National Cheng Kung University, Tainan, Taiwan; King's College, United Kingdom

## Abstract

Annotating protein functions and linking proteins with similar functions are important in systems biology. The rapid growth rate of newly sequenced genomes calls for the development of computational methods to help experimental techniques. Phylogenetic profiling (PP) is a method that exploits the evolutionary co-occurrence pattern to identify functional related proteins. However, PP-based methods delivered satisfactory performance only on prokaryotes but not on eukaryotes. This study proposed a two-stage framework to predict protein functional linkages, which successfully enhances a PP-based method with machine learning. The experimental results show that the proposed two-stage framework achieved the best overall performance in comparison with three PP-based methods.

## Introduction

Various protein functions are essential to diverse biological processes in a living cell. Elucidating these protein functions and linking functional related proteins helps our understanding of the mechanisms of biological systems at the molecular level [1]. With the increasing quantity of sequenced genomes, using biological experiments to identify all functional related proteins is impractical in terms of time and cost. This calls for the development of computational methods.

Various computational methods have been proposed to predict protein functional linkages based on the observation that functionally related proteins have some co-occurrence patterns. Shoemaker and Panchenko have provided a good review of these methods [2]. Gene neighbor and gene cluster methods infer functional linkages from the observation that genes producing interacting proteins usually cluster within a transcription unit, an operon, in the genome [3],[4],[5]. The Rosetta Stone method is based on the pattern that certain interacting proteins have homologues forming a fused protein chain, named a Rosetta Stone protein, in other organisms [6], [7], [8], [9]. Gene neighbor, gene cluster, and the Rosetta Stone method have a common disadvantage that only very limited functional linkages have such specific co-occurrence patterns. Thus, the recent co-occurrence-based methods shifted to phylogenetic profiling (PP), a more general co-occurrence pattern. The basic assumption in PP-based methods is that the co-presence and co-absence of proteins across organisms, the co-evolve pattern, result from the inter-dependence between those proteins [10], [11], [12], [13], [14]. Though PP-based methods delivered satisfactory performance, they have been applied mainly to prokaryotes. This is due to that a collection of organisms, called “reference collection” in the context, is required to construct a PP. Completely sequenced eukaryotic genomes are much less than prokaryotic ones and a prokaryotic reference collection is not suitable for eukaryotic proteins because of the different genomic organizations between prokaryotes and eukaryotes [13], [14].

This study proposed a two-stage framework to analyze protein functional linkages by integrating machine learning (ML) with a PP-based method. ML techniques have been widely used to predict protein relations in many studies [15], [16], [17], [18], in which several techniques have been developed to capture the important features of protein pairs. Shen *et al.* proposed the “conjoint triad” feature, which employs the frequency of three continuous amino acids to encoded protein sequences into feature vectors [15]. They used the support vector machine (SVM) [19] to construct the abstract model of the feature vectors. Guo *et al.* adopted the SVM and proposed an auto cross covariance-based mechanism to encode proteins [16]. Chang *et al.* showed that the features extracted from the protein surface are critical in predicting protein interactions [17]. They used the relaxed variable kernel density estimator (RVKDE) [20] to construct the abstract model. Yu *et al.* adopted the RVKDE and proposed a probability-based mechanism to consider the natural amino acid distribution in encoding protein sequences [18]. The most advantage of the above ML-based methods is that they did not rely on auxiliary information such as localization data and/or interactions from orthologues.

The two-stage framework proposed in this study contained a unique filter to verify the reliability of PPs. The first stage constructs and compares the PPs of the input proteins. Protein pairs with similar PPs are then analyzed by the RVKDE at the second stage. The performance of the proposed framework was compared to three PP-based methods. The effect of the proposed PP verification step and using different reference collections was also evaluated. The experimental results show that the proposed two-stage framework successfully integrated the ML techniques with the PP-based method and achieved the best prediction performance. Furthermore, the performance advantage of the first stage of this study over other PP-based methods reveals that the proposed PP verification step is critical to PP-based methods.

## Materials and Methods

**Figure 1:**
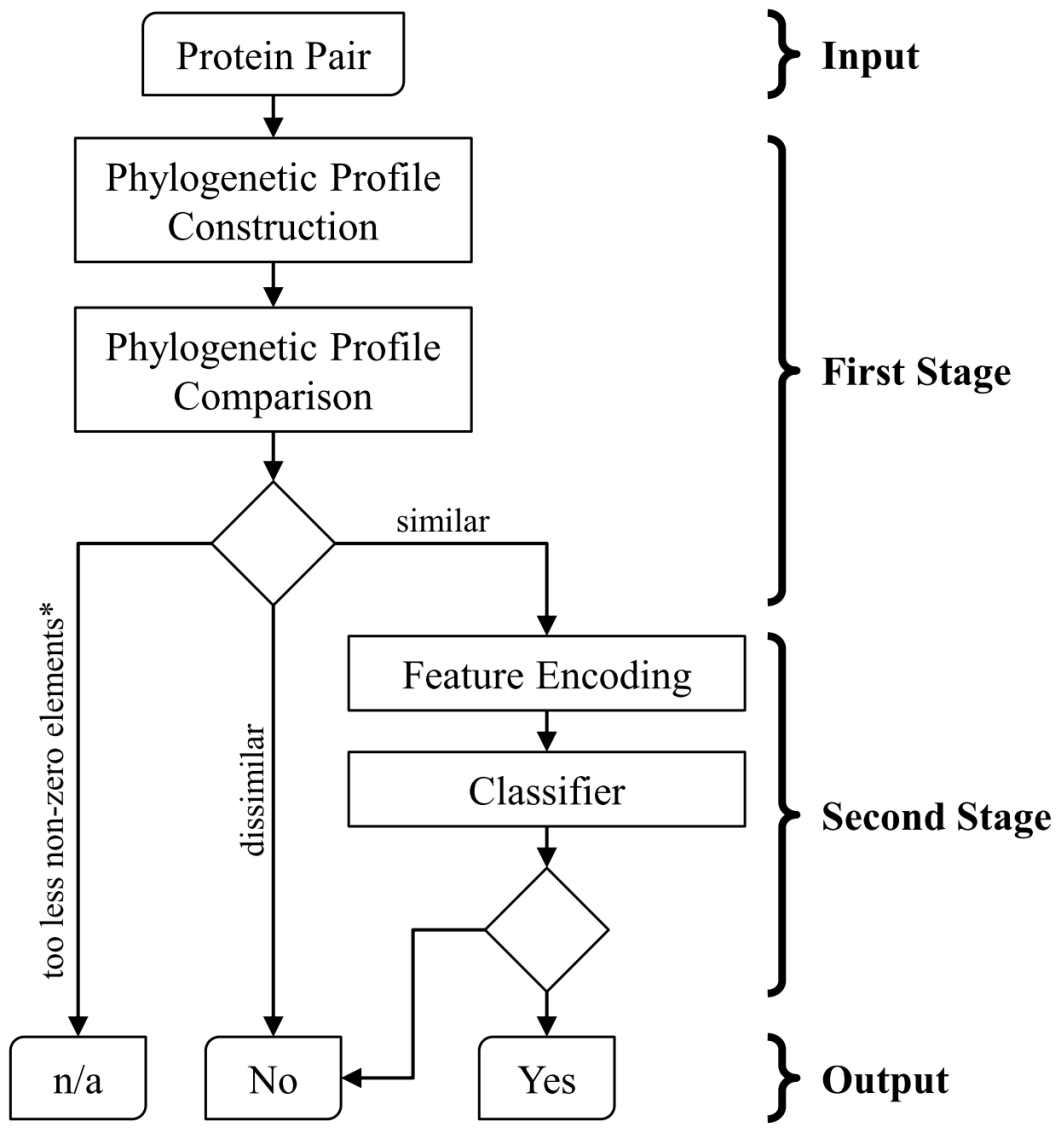
Workflow of the proposed two-stage framework of functional linkage prediction. Given a query protein pair, the first stage constructs and compares their phylogenetic profiles. A unique feature of the first stage is a non-zero filter (marked by an asterisk), which delivers no prediction (denoted as n/a) if either phylogenetic profile contains too less non-zero elements. A pair with similar phylogenetic profiles is submitted to the second stage for the final prediction.

The proposed predictor of protein functional linkages is a two-stage framework (Figure 1). A PP-based approach is employed, where only protein pairs with high phylogenetic similarity are submitted to the second stage, to reduce the data at the first-stage. A unique feature of the first stage of this study to other PP-based approaches is a non-zero filter (marked by an asterisk in Figure 1), which verifies if the phylogenetic similarity is reliable. Next, a ML-based approach is applied on the reduced data for the final prediction. The following subsections describe the details of how to construct and compare the phylogenetic profiles, the non-zero filter, and the adopted features and classifier of the second stage.

### Phylogenetic profile

PP-based methods are based on the observation that genes with similar PPs tend to exist in the same protein complex, biochemical pathway or sub-cellular compartment. Here the PP of a gene is a vector, representing the presence or absence of homologues to that gene across the reference collection. There are two major components in a PP-based method: (i) how to construct a PP of a given gene and (ii) how to determine the similarity of two PPs.

First, the presence or absence of homologues can be determined by sequence alignment scores, such as a BLAST [21] E-value. A protein is considered as “presence” in an organism if the sequence alignment score of the protein between at least one proteins in the organism exceeds a threshold. Such binary vectors were improved as real valued vectors of normalized alignment scores without arbitrarily determining a score threshold. A real valued PP was adopted in this study. Suppose that there is a collection of *n* reference organism used to build the PP of a query gene. The first step is to compare the open reading frame (ORF) of the query gene to all ORFs of the *n* reference organism using BLAST. The best bit score of the query gene *a* and all ORFs of a reference organism *b* is used to measure the presence of *a* in *b*, called “*S*-value of gene *a* and organism *b*” and denoted as *S_ab_*. As non-homologous genes have certain chance to form an alignment with the bit score higher than 50 [22], *S*-value is trimmed to zero if it is lower than 50. Considering that the bit score depends on the sequence of *a*, *S*-value is further normalized as *R*-value by the following equation:



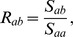
where *S_aa_* is the score obtained by aligning *a* to itself. The *n*-dimensional vector of *R*-values obtained by BLASTing a gene to *n* reference organisms represents the phylogenetic profile of that gene. In addition the non-zero *R*-values of all genes of the query organism to a reference organism are normalized by dividing the average score. This procedure prevents the similarity between two phylogenetic profiles of two genes being dominated by a few large *R*-values obtained from phylogenetically close organisms.

Second, any similarity/distance function, such as the cosine similarity or Euclidean distance [23], of vectors can be used to define the similarity of PPs. Enault *et al.* have examined four widely used distance functions and concluded that the inner product is a good indicator [12]. In this study, the similarity between two genes *i* and *j* is defined as follows:



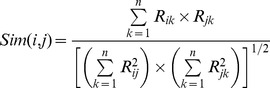
.

### Non-zero filter

The non-zero filter (marked by an asterisk in Figure 1) is designed for rare genes that have homologues in only a few reference organisms. Accordingly, the PP of a rare gene contains only a few non-zero values. The similarity between two rare genes highly depends on these non-zero values. This condition is similar to using a small reference collection, thereby reduces the reliability of the calculated similarity. An extreme example is that if two genes present in only a reference organism, a high similarity of their PPs may result from the similar phylogenetic characteristic or the rareness. Thus, this study introduced a threshold, denoted as *nz*, to solve this problem. If the number of non-zero elements of either PP vector of two genes *i* and *j* are less than *nz*, their PP similarity is regarded as unreliable based on the adopted reference collection.

### Feature encoding

The second stage of this work encodes a gene pair into a feature vector and then invokes a classifier to perform the prediction. This subsection describes the feature encoding while the next subsection describes the classification algorithm.

The used feature set considers the conjoint triads observed in the protein sequence [15]. A conjoint triad regards three continuous residues as a unit. Each gene pair is then encoded by concatenating the two feature vectors of the two individual genes. However, considering all 20^3^ conjoint triads requires a 16000-dimensional feature vector to encode a gene pair, which is too large for contemporary classifiers to analyze. Thus, Shen *et al.* clustered the 20 amino acid types into seven groups based on the dipole strength and side chain volumes to reduce the dimensions of the feature vector. The seven amino acid groups are listed in Table 1.

**Table 1:** Amino acid groups adopted in this study.

Group no.	Amino acids
1	Ala, Gly, Val
2	Ile, Leu, Phe, Pro
3	Tyr, Met, Thr, Ser
4	His, Asn, Gln, Trp
5	Arg, Lys
6	Asp, Glu
7	Cys

This table follows the Shen et al.’s work [15].

**Figure 2:**
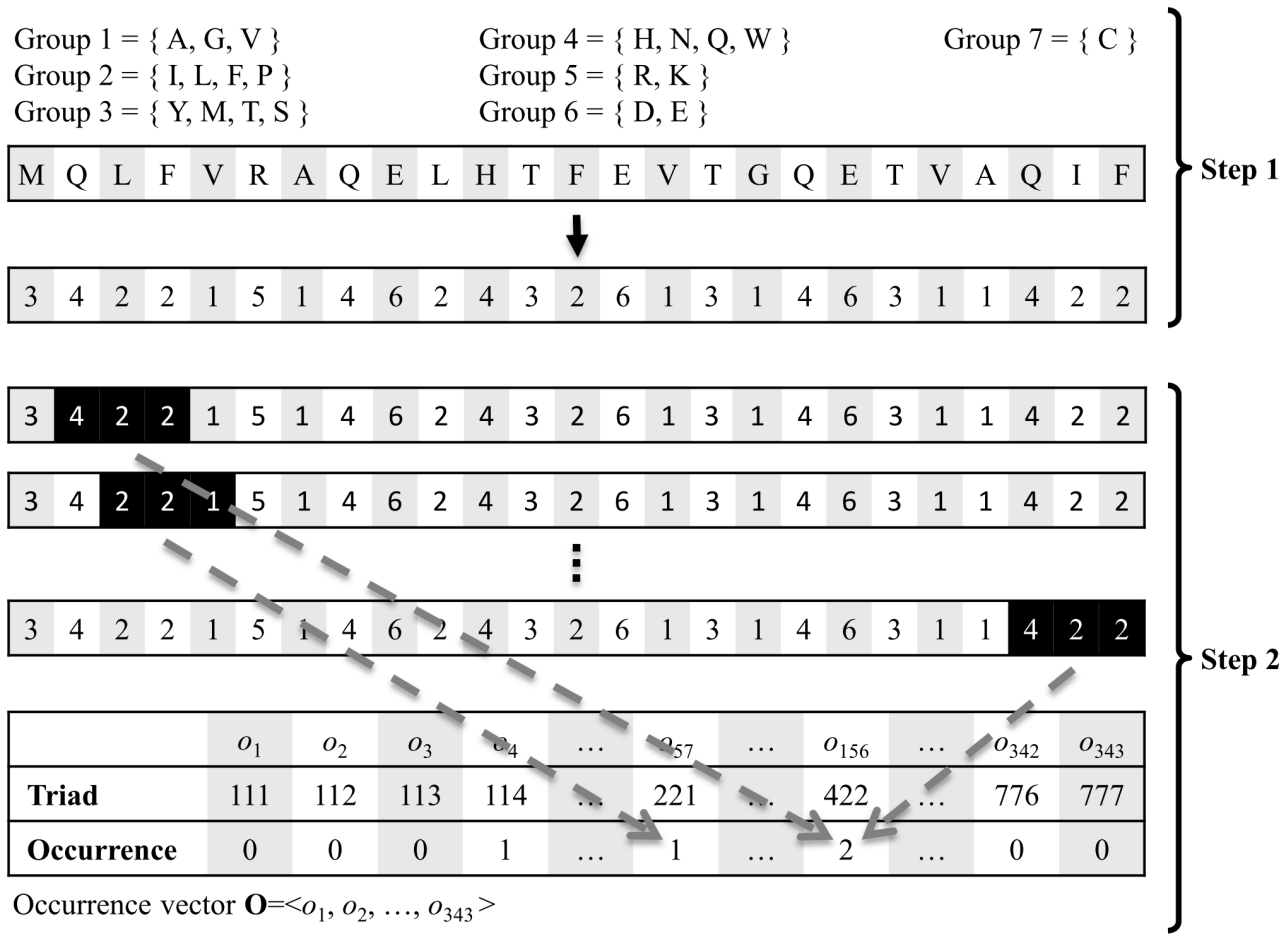
Schematic diagram of encoding a protein sequence into a feature vector. Step 1: Transform the amino acid sequence into the group sequence. Step 2: Scan the group sequence and count the triads in the occurrence vector **O**.

The process of encoding a protein sequence is shown in Figure 2. First, the protein sequence is transformed into a sequence of amino acid groups. Then the triads are scanned along the sequence of amino acid groups. Each scanned triad is counted in an occurrence vector, **O**. Each element *o_i_* in **O** represents the number of the *i*-th type of triad observed in the sequence of amino acids groups. Accordingly, each protein sequence is represented as a 343-dimentional occurrence vector. For a protein pair, the two vectors of both protein sequences are concatenated to form a 686-dimensional feature vector.

### Relaxed variable kernel density estimator

This study adopts the relaxed variable kernel density estimator (RVKDE) to construct the abstract model of the encoded feature vectors. The RVKDE has been shown achieved excellent performance in predicting protein interactions [18]. It sacrificed a slight prediction performance but largely reduces the execution time by 1% compared to the well-known support vector machine (SVM). One main distinctive feature of the RVKDE is that it features an average time complexity of *O*(*n*log*n*) for carrying out the training process, where *n* is the number of instances in the training set. The concept of the RVKDE is described as follows.

Let 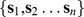 be a set of instances randomly and independently taken from the distribution governed by *f*_x_ in the *m*-dimensional vector space. Then the probability density function of *f*_x_ at point **v** is estimated by the following equation:



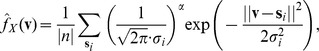
where

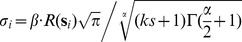

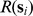
 is the maximum distance between 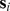 and its ks nearest training samples;

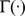
 is the Gamma function [24];
4. *α*, *β* and *ks* are parameters to be set either through cross validation or by users.

When using the RVKDE to predict functional linkages, two probability density functions are constructed to approximate the distributions of functional related and unrelated protein pairs in the training set. A query protein pair (represented as the feature vector **v**) is predicted to the class that gives the maximum value among the two likelihood functions defined as follows:



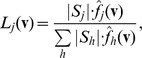
where 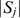 is the number of class-*j* training instances**,** and 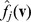 is the probability density function corresponding to class-*j* training samples. In this study, *j* is either “related” or “unrelated”. In order to improve the efficiency, the RVKDE includes only *kt* nearest training samples of **v** when computing 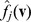. The parameter *kt* is set either through cross-validation or by users.

## Results

This study conducted several experiments to evaluate the proposed two-stage predictor of protein functional linkages. The first subsection describes the data collection of these experiments. The second subsection shows the performance of the two-stage predictor as well as of individual stages. The performance was also compared with three PP-based predictors in the third subsection. Finally, the last subsection discusses the suitable *nz* thresholds of the non-zero filter using different reference collections.

### Data collection

This study retrieved 6,290 genes and 92 pathways of *Saccharomyces cerevisiae* from the Kyoto Encyclopedia of Genes and Genomes (KEGG) database released at May 1, 2012 [25]. A protein whose gene sequence is longer than 150 nt and protein sequence contains only proteinogenic amino acids was preserved. Proteins that participate none of the collected 92 pathways were excluded. The final collection of *S. cerevisiae* in this study consisted of 1,466 proteins, which form 1,073,845 pairs. Two proteins that participate at least a common pathway was defined as a positive pair, otherwise a negative pair. There were 224,376 positive and 849,469 negative pairs in the *S. cerevisia* collection. In this study, *S. cerevisia* was used as the query organism, namely the evaluation organism, of which the prediction performance was used to evaluate methods.

In addition to an evaluation organism, the first stage of the proposed framework requires a reference collection to construct PPs. This study used the 829 prokaryotes and 132 eukaryotes in the KEGG database to compile a prokaryotic and a eukaryotic reference collection, respectively. *S. cerevisiae* was not included in the eukaryotic reference collection. In addition, a third reference collection consisting of only the 220 prokaryotic organisms released before 2006 was compiled for a fair comparison with other approaches of PPI prediction. The newest strain was used if multiple strains were available for an organism. In these reference organisms, only the gene and protein sequences were required. Since the functional linkage information of the reference organisms was not used, these organisms were not training data in machine learning. For a fair comparison, the reference collection of 220 prokaryotes was used in the second and third subsections. The other two reference collections were used in the fourth subsection to analyze the effect of using different reference collections.

Finally, the second stage of the proposed framework needs a training organism of which the functional linkage information is required. This study retrieved 4,493 proteins and 106 pathways of *Escherichia coli* from the KEGG database. After applying the same filters as the query organism, the training organism of this study consisted of 1,355 *E. coli* proteins, which form 217,155 positive and 700,180 negative pairs.

All the parameters of the RVKDE (*α*, *β*, *ks* and *kt*) are decided using five-fold cross validation on the training data. The best parameter combination was selected by using a grid search approach to maximize the *F-measure* on the *E. coli* data.

### Contribution of each stage

This subsection reports the performance of the proposed two-stage approach as well as the performance of the first and the second stage individually. Conventional PP-based methods need to determine a similarity threshold, where protein pairs with similarity higher than the threshold are predicted as functional related proteins. In this study, the PP-based first stage was a filter to reduce the input data of the second stage. The threshold was selected to preserve the most reliable data while making the second stage computationally applicable. In this experiment, an extremely loose threshold was used—only protein pairs with zero similarity were filtered—since the resulting data of 2,163 protein pairs can be processed by the RVKDE in a minute. The non-zero filter was also set to an extremely loose threshold *nz*  =  1, namely only PPs with all zeros were filtered. As will be illustrated in the fourth subsection, the non-zero filter, though it was very loose, did help the prediction performance.

The preparation of using either stage as an individual predictor was similar to the procedure of the two-stage framework except that (i) the individual first stage used the PP similarity, instead of the likelihood reported by the RVKDE, to rank protein pairs and (ii) the input data of the individual second stage was selected randomly from the pairs that passed the non-zero filter while preserving the quantities of positive and negative samples equal to those selected by the first stage. The two-stage, the individual first stage and the individual second stage are respectively denoted as Pred_both_, Pred_1st_ and Pred_2nd_ in the context. Since the preparation of Pred_2nd_ involved randomness, the prediction process of Pred_2nd_ was repeated ten times to alleviate bias. When regarding the second stage as an individual predictor, an advantage of the Pred_2nd_ over the Pred_both_ is that its coverage is not limited by the Pred_1st_. The coverage of the Pred_both_ and Pred_1st_ was less than 0.5% (1,052/224376, where only 1,052 of the 2,163 protein pairs are functional related). However, ML techniques are computationally infeasible for genome-wide prediction with contemporary computers. A sampling procedure, usually random sampling in practice, must be performed before applying ML techniques. Our results show that the Pred_1st_ is suitable for this procedure.

**Figure 3:**
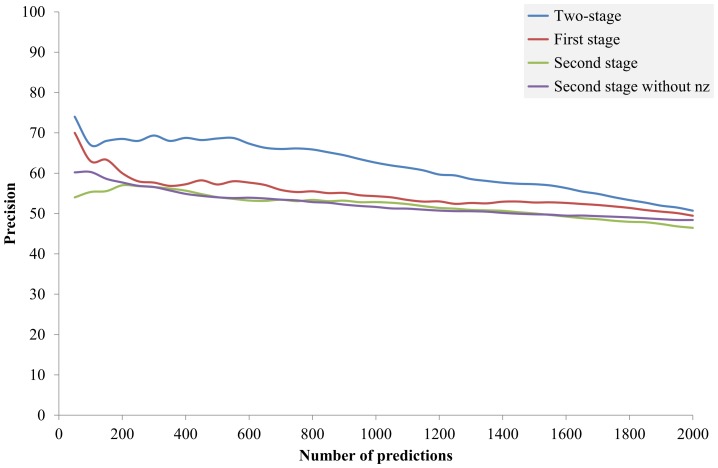
Performance of the proposed two-stage approach and the individual stages. The *y*-axis is the ratio of correct predictions predicted in the top *n* predictions (*x*-axis).

Figure 3 depicts the precision, as measured by positive predictive value (PPV), of the 2000 highest ranked predictions made by the Pred_both_ (the blue line), Pred_1st_ (the red line) and Pred_2nd_ (the purple line). The green line indicates the performance of Pred_2nd_ without the non-zero filter, which is used in the “Effect of the non-zero filter” section. The Pred_both_ achieved the best performance, especially in the high-precision area. Compared to the Pred_1st_, the Pred_both_ had an advantage of precision by >10% at the top 250 predictions and this advantage remained >5% until the top 1,350 predictions. The diminishing advantage is reasonable since the Pred_both_ used the Pred_1st_ as a filter to obtain a smaller data of the 2,163 samples of the query organism. Their performances were getting close as the number of predictions approaching 2,163. Compared to the Pred_2nd_, the Pred_both_ outperformed by 13.8–2.3% from top 50 to 2000 predictions. The Pred_1st_ was better than the Pred_2nd_ in this experiment. A possible explanation is that the Pred_1st_ utilized 220 reference organisms while the Pred_2nd_ utilized only *E. coli*. Another observation is that the Pred_2nd_ was more stable than the Pred_1st_. In summary, the proposed two-stage yielded better performance than either stage and was as stable as the Pred_2nd_. It means that this study has combined two different types of approaches successfully.

### Comparison with existing methods

This subsection compares the proposed two-stage framework to three PP-based methods [10], [11], [12]. The first PP-based method is the binary PP—the occurrence pattern of a protein to an organism is either 0 or 1—optimized for prokaryotes by Sun and colleagues [11]. The second PP-based method is the quantized PP—the occurrence pattern is discretized into one of 11 bins—proposed by Date and Marcotte [10]. This method has been shown effective in both *E. coli* and *S. cerevisiae*. The third PP-based method is the continuous PP—the occurrence pattern is a real number—proposed by Enault and colleagues [12]. The first stage of this study was improved from the third PP-based method by introducing the non-zero filter.

**Figure 4:**
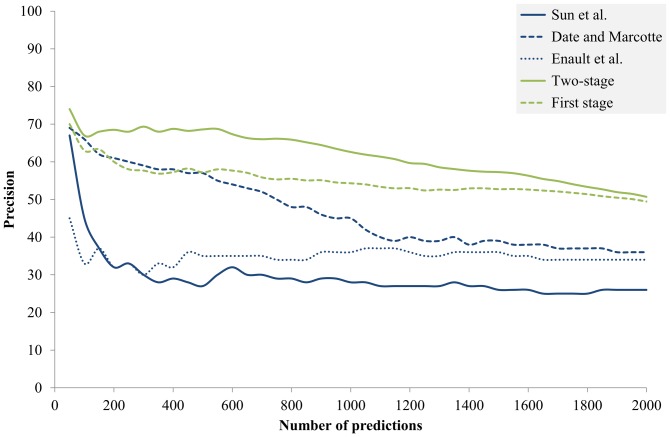
Comparison of the proposed two-stage approach to three PP-based techniques. The *y*-axis is the ratio of correct predictions predicted in the top *n* predictions (*x*-axis).

Figure 4 shows the performance of the proposed two-stage approach and the compared methods. The preparation procedure of the PP-based methods was identical to that for the Pred_1st_ described in the previous subsection. When comparing the Pred_1st_ (the green dashed line in Figure 4) with the three PP-based methods, the Pred_1st_ delivered comparable performance to Date and Marcotte in the high-precision area and achieved the best overall performance. The Pred_1st_ was improved from the Enault *et al*. method by introducing the non-zero filter. The notable performance difference between the Pred_1st_ and the Enault *et al*. method reveals the importance of the proposed non-zero filter. The effect of the non-zero filter will be elaborated in the next subsection. In summary, the first stage of the proposed method was better than all the compared methods. Furthermore, the proposed method achieved the best performance without depending on the number of predictions. It stably yielded >10% precision advantage over the compared methods in the range of top 300–1100 predictions.

Table 2 and Table 3 show the area under curves (AUCs) at specific recalls (*i.e.*, coverage) of the proposed two-stage approach and the compared methods. The AUCs in Table 2 were calculated in the recall (*x*)-precision (*y*) plane. This plane is slightly different to Figure 4 of which the *x*-axis is number of predictions. The AUCs in Table 2 were extremely low because of the adopted recalls, which were chosen according to the <0.5% coverage of the Pred_both_ and Pred_1st_. Table 3 shows the adjusted AUCs by dividing the AUC that a perfect predictor can achieve at the corresponding recall. Namely, Table 3 shows the AUC ratios of methods to a perfect predictor. The results of the two tables indicate that the Pred_both_ achieved the best performance and the Pred_1st_ achieved the second best performance, which were consistent with Figure 4.

**Table 2:** Area under curve (AUC) comparison at specific recalls.

Recall	Sun et al.	Date and Marcotte	Enault et al.	Two-stage	First stage	Second stage
0.001	0.0003	0.0006	0.0003	**0.0007**	0.0006	0.0006
0.002	0.0006	0.0012	0.0007	**0.0014**	0.0012	0.0011
0.003	0.0008	0.0017	0.0010	**0.0020**	0.0018	0.0017
0.004	0.0011	0.0022	0.0014	**0.0027**	0.0023	0.0022
0.005	0.0014	0.0025	0.0017	**0.0034**	0.0029	0.0027

The AUCs are calculated in the recall (*x*)-precision (*y*) plane. The best precision at a specific recall is highlighted in bold.

**Table 3:** Adjusted area under curve (AUC) comparison at specific recalls.

Recall	Sun et al.	Date and Marcotte	Enault et al.	Two-stage	First stage	Second stage
0.001	0.2818	0.6100	0.3177	**0.6778**	0.6040	0.5728
0.002	0.2838	0.6020	0.3258	**0.6783**	0.5986	0.5657
0.003	0.2826	0.5836	0.3358	**0.6795**	0.5911	0.5571
0.004	0.2784	0.5518	0.3416	**0.6781**	0.5843	0.5497
0.005	0.2749	0.5190	0.3430	**0.6720**	0.5761	0.5421

The adjusted AUCs are the AUCs in Table 2 divided by the AUC that a perfect predictor can achieve at the corresponding recall. The best precision at a specific recall is highlighted in bold.

### Effect of the non-zero filter

Comparing the Pred_1st_ and the Enault *et al.* method in Figure 4 reveals the effect of the non-zero filter to the first stage. The Pred_1st_ was improved from the Enault *et al*. method by introducing the non-zero filter. Surprisingly, the Pred_1st_ delivered the best performance while the Enault *et al*. method delivered the worst performance in the high-precision area in Figure 4. By manually examining the generated PPs, we concluded that some PP pairs had high similarity in the Enault *et al.* method only because of having too many zeros. Thus, their similarities depended only on a few reference organisms and were less reliable. This observation explains the good performance of the Pred_1st_, whose non-zero filter was designed to solve this problem. On the other hand, comparing the “Second stage” (the purple line) and the “Second stage without nz” (the green line) in Figure 3 reveals the effect of the non-zero filter to the second stage. The results show that the non-zero filter did not obviously contribute to the second stage. The performance of the second stage with the non-zero filter in the top 200 predictions was even worse than then that without the non-zero filter. This is reasonable since that the non-zero filter only helped to extracting protein pairs with reliable PPs. The extracted pairs are not necessarily more functional related than those with unreliable PPs.

**Figure 5:**
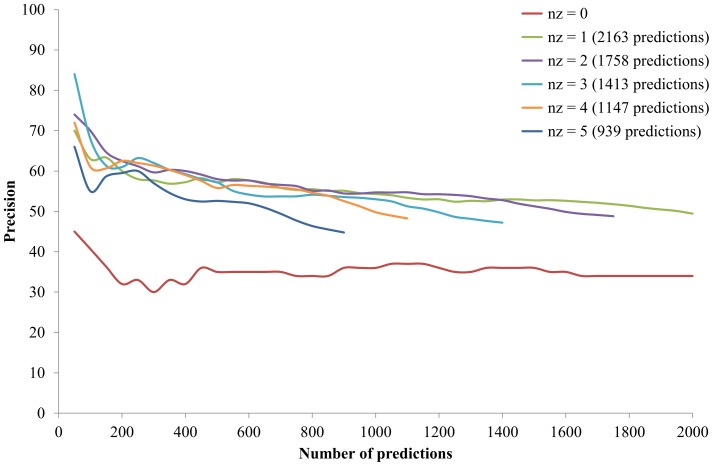
Performance of using different non-zero (*nz*) thresholds. The *y*-axis is the ratio of correct predictions predicted in the top *n* predictions (*x*-axis).

However, to choose a proper threshold of *nz* raises a new problem. This subsection further elaborates whether different *nz* thresholds largely affect the prediction performance. Figure 5 shows the performance of the Pred_1st_ using different *nz* thresholds. Using a larger *nz* made fewer predictions (from 2,163 to 939 predictions in Figure 5) as less protein pairs passed the non-zero filter. In theory, using a larger *nz* requires PPs having more non-zero elements for similarity calculation and achieves a higher precision. This tendency can be observed from *nz*  =  0 to *nz*  =  3. However, increasing the *nz* threshold to four and five decreased the prediction performance. The result that the best *nz* threshold is small indicates that the PPs of many proteins in the query organism did contain many zero elements. This could be resulted from that (i) the reference collection was small or (ii) the reference collection was dissimilar to the query organism. To further elucidate this phenomenon, the prediction performances of using different *nz* thresholds were also analyzed on two other reference collections.

**Figure 6:**
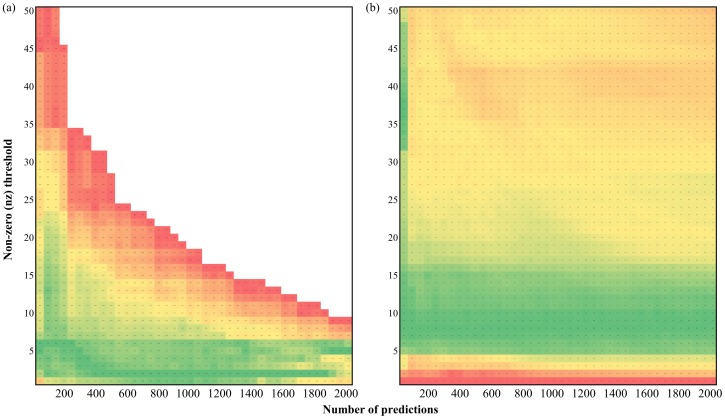
The relationships among the number of predictions (*x*-axis), the non-zero thresholds (*y*-axis) and the prediction performance (color) with different reference collections of (a) 829 prokaryotes and (b) 132 eukaryotes. Since different applications might require different number of predictions, the colors of a specific abscissa were normalized from red to green. Thus, one can quickly identify the best non-zero threshold under a specific number of predictions.

**Figure 7:**
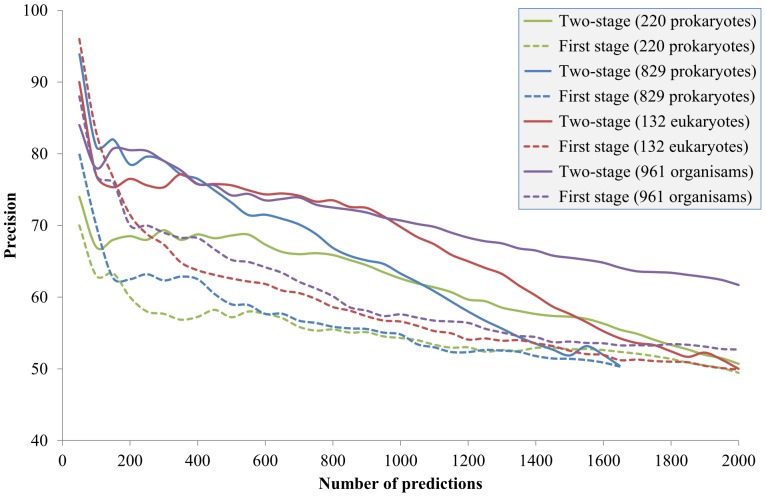
Performance using different reference collections. The *y*-axis is the ratio of correct predictions predicted in the top *n* predictions (*x*-axis).

First, the larger prokaryotic reference collection of 829 prokaryotes was used. Figure 6(a) shows the best *nz* threshold using this reference collection. The best *nz* threshold was larger than that using the reference collection of 220 prokaryotes, especially in the high-precision (namely, few-prediction) region. This echoes that the size of the reference collection does affect the suitable *nz* thresholds. Figure 6(a) also reveals how many non-zero elements are required to represent the PP of a *S. cerevisiae* protein based on the prokaryotic reference collection. If researchers focus on the top 150 predictions (>80% precision, see the blue solid line in Figure 7), the best *nz* threshold is close to five and any *nz* thresholds in the range of 20–30 are fine; if researchers focus on the top 150–400 predictions (75–80% precision), the best *nz* threshold decreases from five to two and any *nz* thresholds in the range of 1–10 are fine. As more predictions are desired, the *nz* threshold should be set to near two and not exceeding five.

In this study, a reference collection of 132 eukaryotes was also compiled. In comparison with the reference collection of 220 prokaryotes, the reference collection of 829 prokaryotes is a more comprehensive collection while the reference collection of 132 eukaryotes is a collection more similar to the query organism. Figure 6(b) shows the best *nz* threshold using the eukaryotic reference collection. The results indicate that 5–15 non-zero elements are required to represent the occurrence pattern of a *S. cerevisiae* gene constructing from prokaryotes. The best *nz* threshold is consistent without depending on the number of predictions. The best *nz* threshold was more stable than that using the previous two prokaryotic reference collections. Furthermore, with the eukaryotic reference collection, the Pred_1st_ achieved >90% and >80% precision in the top 50 and 100 prediction, respectively. This performance is better than that using the prokaryotic reference collections. Hence, the prediction performance of the proposed method was re-evaluated using the eukaryotic reference collection (Figure 7).

The performance of the PP-based first stage using 132 eukaryotes (the red dashed line Figure 7) was superior over those using prokaryotes (the green and blue dashed lines in Figure 7) as the reference collection. On the other hand, the performance using 829 prokaryotes (the blue dashed line) was not obviously better than that using 220 prokaryotes (the green dashed line). This suggests that PP-based methods rely more on the similarity to the query organism, rather than the size, of the reference collection. Combining 829 prokaryotes and 132 eukaryotes as the reference collection (961 organisms) delivered slightly better overall performance then that using 132 eukaryotes, except in the very top predictions. This suggests that enlarging the reference collection helps the prediction of protein pairs with relatively low phylogenetic similarity. Finally, changing the reference collections influences not only the prediction accuracy but also the number of predictions (*i.e.* pairs that pass the non-zero filter and the first stage) that the predictor can deliver. Table 4 shows the number of pairs that passed the non-zero filter and the first stage, namely number of pairs with reliable and similar PPs, obtained using different reference collections.

**Table 4:** Number of pairs that passed the non-zero filter and the first stage using different reference collections.

Reference collection (the best *nz* value)	Number of pairs
220 prokaryotes (1)	2,163
829 prokaryotes (2)	1,651
132 eukaryotes (8)	263,779
961 organisms (7)	268,397

This table reveals the number of pairs with reliable and similar PPs using different reference collections.

The previous studies concluded that PP-based approaches are not capable for eukaryotes [12], [13]. Furthermore, they found that using eukaryotes as the reference collection led to worse performance than using prokaryotes due to the insufficiency of completely sequenced eukaryotes. However, through introducing the proposed non-zero filter, this study slightly refined the conclusion of previous studies to that PP-based methods are promising for eukaryotes based on currently available eukaryotic genomes with an appropriate mechanism to verify the reliability of PPs.

## Conclusions

This study proposed a two-stage predictor of protein functional linkages, which successfully integrates machine learning techniques with phylogenetic profiling-based methods as well as introduces a non-zero filter to enhance the reliability of phylogenetic similarity. The experimental results show that the proposed two-stage framework achieved good performance and preserved the advantages of both categories of techniques: (i) high performance in the top predictions of phylogenetic profiling and (ii) stable performance of machine learning. In addition, the proposed non-zero filter has been shown that phylogenetic profiling-based methods are promising for eukaryotes based on currently available eukaryotic genomes. The discovery of this study helps analyzing protein functional linkages and encourages developing hybrid framework in the future.
